# Health Researchers’ Use of Social Media: Scoping Review

**DOI:** 10.2196/13687

**Published:** 2019-11-13

**Authors:** Justine Dol, Perri R Tutelman, Christine T Chambers, Melanie Barwick, Emily K Drake, Jennifer A Parker, Robin Parker, Eric I Benchimol, Ronald B George, Holly O Witteman

**Affiliations:** 1 Dalhousie University Faculty of Health Halifax, NS Canada; 2 IWK Health Centre Centre for Pediatric Pain Research Halifax, NS Canada; 3 Dalhousie University Department of Psychology and Neuroscience Halifax, NS Canada; 4 Dalhousie University Department of Pediatrics Halifax, NS Canada; 5 The Hospital for Sick Children SickKids Research Institute Child Health Evaluative Sciences Toronto, ON Canada; 6 University of Toronto Faculty of Medicine Toronto, ON Canada; 7 University of Toronto Dalla Lana School of Public Health Toronto, ON Canada; 8 Dalhousie University WK Kellogg Health Sciences Library Halifax, NS Canada; 9 University of Ottawa Faculty of Medicine Department of Pediatrics Ottawa, ON Canada; 10 University of Ottawa Faculty of Medicine School of Epidemiology and Public Health Ottawa, ON Canada; 11 University of California San Francisco Department of Anesthesia and Perioperative Care San Francisco, CA United States; 12 Laval University Faculty of Medicine Department of Family and Emergency Medicine Quebec, QC Canada; 13 Laval University Faculty of Medicine Office of Education and Professional Development Quebec, QC Canada; 14 CHU de Québec-Université Laval Quebec, QC Canada; 15 Ottawa Hospital Research Institute Ottawa, ON Canada

**Keywords:** health, social media, review

## Abstract

**Background:**

Health researchers are increasingly using social media in a professional capacity, and the applications of social media for health researchers are vast. However, there is currently no published evidence synthesis of the ways in which health researchers use social media professionally, and uncertainty remains as to how best to harness its potential.

**Objective:**

This scoping review aimed to explore how social media is used by health researchers professionally, as reported in the literature.

**Methods:**

The scoping review methodology guided by Arksey and O’Malley and Levac et al was used. Comprehensive searches based on the concepts of health research and social media were conducted in MEDLINE, EMBASE, CINAHL, PsycINFO, ERIC, and Web of Science databases, with no limitations applied. Articles were screened at the title and abstract level and at full text by two reviewers. One reviewer extracted data that were analyzed descriptively to map the available evidence.

**Results:**

A total of 8359 articles were screened at the title and abstract level, of which 719 were also assessed at full text for eligibility. The 414 articles identified for inclusion were published in 278 different journals. Studies originated from 31 different countries, with the most prevalent being the United States (52.7% [218/414]). The health discipline of the first authors varied, with medicine (33.3% [138/414]) being the most common. A third of the articles covered health generally, with 61 health-specific topics. Papers used a range of social media platforms (mean 1.33 [SD 0.7]). A quarter of the articles screened reported on social media use for participant recruitment (25.1% [104/414]), followed by practical ways to use social media (15.5% [64/414]), and use of social media for content analysis research (13.3% [55/414]). Articles were categorized as *celebratory* (ie, opportunities for engagement, 72.2% [299/414]), *contingent* (ie, opportunities and possible limitations, 22.7% [94/414]) and *concerned* (ie, potentially harmful, 5.1% [21/414]).

**Conclusions:**

Health researchers are increasingly publishing on their use of social media for a range of professional purposes. Although most of the sentiment around the use of social media in health research was celebratory, the uses of social media varied widely. Future research is needed to support health researchers to optimize their social media use.

## Introduction

Health researchers are using social media in a professional capacity [1]. Defined as interactive internet-based applications that enable users to share information, network, and collaborate on the Web [2], well-known examples of social media platforms include Twitter, Facebook, and YouTube. Although social media use has historically been met with skepticism in the health research community [3,4], several major science journals now publish articles endorsing the relevance of social media for researchers [5,6]. The applications of social media for health research are vast; researchers report using social media to post content and keep abreast of advancements in their field (eg, new publications), to network with colleagues and knowledge users (eg, hashtag communities and journal clubs), to conduct research (eg, participant recruitment and social media as a dissemination or data collection tool), and for academic promotion [5,7,8].

Researchers are now actively encouraged to utilize social media in their research, with social media engagement being increasingly recognized by institutions as an important evaluation criterion for promotion and tenure [9]. Social media in the form of live-tweeting is increasingly present at academic conferences, where delegates share content using a specified conference hashtag [10], and many journals now have dedicated editors or committees who promote newly published scientific papers via social media [11]. Evidence is rapidly accumulating on the scholarly impact of social media activities. Some studies have shown that the promotion of research articles over social media channels significantly increases their reach, as evidenced by more article views, downloads, and citations [12-15], though evidence from a randomized controlled trial at a single journal on 243 articles found no difference in page views [16]. Social media is also influencing how we disseminate our research. Many journals have adopted the use of *visual abstracts*, a simple visual representation of the key findings, designed to enhance social media dissemination of health research [17] or encouraging the submission of a tweet to be sent out when the article is published.

Previous reviews have summarized the literature on researchers’ use of social media platforms [7] in specific areas of health [18] and for certain purposes [19]. A scoping review was conducted in 2013 on social media use by health professionals and trainees, rather than health researchers [20]. However, there is no evidence synthesis of the ways in which health researchers, as a specific population group, are using social media across platforms, and there remains uncertainty about how to best harness the potential of this medium in health research. A recent review on the use of Twitter to drive research impact concluded that “advice and guidance on the use of social media for research studies is not well understood or exploited by the research community” [21]. Therefore, the objective for this scoping review was to map the literature on the ways in which health researchers report on their use of social media from the existing literature.

## Methods

### Overview

Health researchers’ use of social media was explored using a scoping review guided by the methodology of Arksey and O’Malley [22] and Levac et al [23]. A scoping review protocol was created to guide the process and is available from the corresponding author upon request. This paper adheres to the Preferred Reporting Items for Systematic Reviews and Meta-Analyses (PRISMA) extension for scoping reviews [24].

### Search Strategy

An experienced information specialist (RP) developed comprehensive search strategies for 6 electronic citation databases: MEDLINE (OvidSP, 1946 to May 2018), EMBASE (Elsevier, 1947 to May 2018), CINAHL (EBSCOHost, 1971 to May 2018), PsycINFO (EBSCOHost, 1967 to May 2018), ERIC (ProQuest, 1966 to May 2018), and Web of Science Core Collection (Clarivate Analytics, 1900 to May 2018). The search strategies utilized index terms, where appropriate, and free text terms to capture the following concepts: (1) social media, including both general terms and specific platform names and terms (eg, Twitter, tweet, Facebook, Snapchat, YouTube); (2) research or researchers; and (3) health or medicine descriptors were added in the non–health discipline databases only (ie, ERIC and Web of Science). The search approach balanced comprehensiveness with precision by including and exploring the general index terms such as *research*, *scientist*, and *social media*, while using adjacency operators to combine the free text search terms. Applying adjacency (or proximity) operators to the text word terms restricts results to those where a relational association exists in the title or abstract text between social media and the research context or researchers. Before finalizing, the searches were checked for sensitivity and relevance and peer reviewed for accuracy and consistency. For the full search strategies in all databases, see Multimedia Appendix 1.

### Inclusion and Exclusion Criteria

Articles were determined eligible for inclusion if they discussed the use of social media by health researchers, including but not limited to use of social media for recruitment, data mining, social media initiatives or campaigns, hashtag communities, and journal clubs. Articles could be from health researchers at any stage of their career (trainee to faculty member) and across any types of health research (policy, services, outcomes, medical, and basic). Articles were included if authors studied or commented on the uses, benefits, or limitations of social media for health researchers. All article types were included, including dissertations, conference abstracts, and opinion pieces, with the exception of systematic or scoping reviews, books, or book chapters. All articles published since 2000 were included given the rapid advancement of social media and the limited social media literature available before 2000.

Articles were excluded if they were written in a language other than English or if they focused on health care providers’ social media use outside of research, organizational, private sector (eg, publishers), or funding agency context. Systematic reviews, scoping reviews, books, and book chapters were excluded. Articles were excluded if they only used social media as a method of recruitment without reporting the uses, benefits, or limitations in relation to their study. Articles were also excluded if they solely reported on the secondary analysis of research output of health researchers, such that a study analyzing research impact data would be excluded (eg, Altmetric reports of a published article).

### Data Extraction

**Figure 1:**
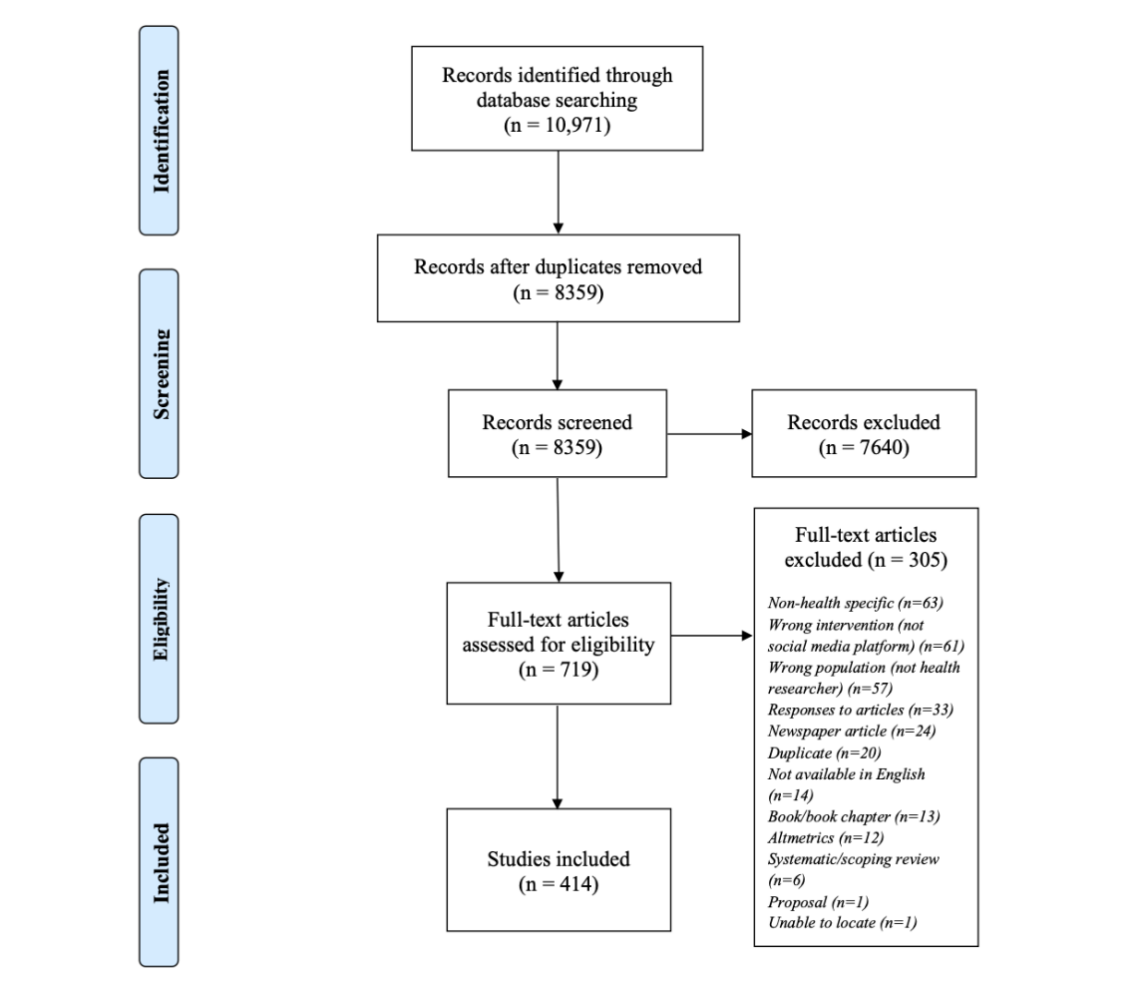
Preferred Reporting Items for Systematic Reviews and Meta-Analyses for Scoping Reviews flow diagram of the search and study selection process.

The screening process was conducted using the PRISMA extension for scoping reviews (Figure 1) [24,25]. At least two reviewers (JD, PRT, JAP, and EKD) screened the titles and abstracts using Covidence [26]. One reviewer (JD) extracted all data from the included articles using abstract data when available with a standardized Google form that was approved by the project team. Extracted data included article characteristics (year of publication, journal, country of first author, article type, health discipline, and academic affiliation), area of health research, social media platform, and preidentified categories related to the purpose for social media use (eg, recruitment and content analysis).

As the internet is now a major source of health information, user sentiment regarding this growth within the medical, sociological, and popular literature has been categorized by Nettleton et al [27] as *celebratory* (offering opportunities of empowerment and engagement for individuals), *contingent* (recognizes the potential positive empowerment yet acknowledges potential limitations), or *concerned* (identified as potentially dangerous owing to unknown quality or reliability of Web-based information). These categories of user sentiment were applied to the identified articles in this review by the reviewer who extracted the data.

For all variables but one, a single response option was selected that best characterized the article. The exception was made for social media platform used, whereby all platforms mentioned in an article were selected. Articles describing general social media use were tagged as such. Extracted data were exported from Google Forms into Microsoft Excel to be cleaned before being imported into IBM SPSS version 22.0 for analysis using descriptive statistics (eg, totals and percentages).

## Results

### Study Selection

On the basis of the initial search, 8359 articles were identified after duplicates were removed. At the title and abstract screening stage, 7640 articles were excluded. A total of 719 articles were screened as full text, and a further 305 articles were excluded for reasons outlined in Figure 1. One reviewer (JD) extracted all data from the 414 included articles.

### Article Characteristics

**Figure 2:**
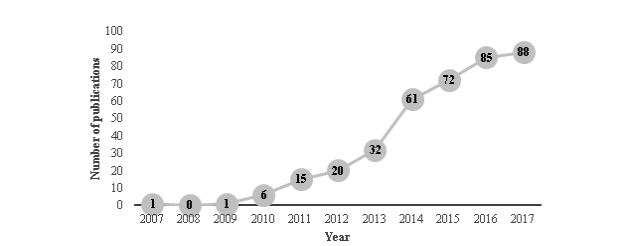
Number of publications by year.

The study identified 414 unique articles across 278 different journals. The number of articles published on health researchers’ use of social media has increased significantly over time (see Figure 2), ranging from 1 publication in 2007 to 88 in 2017. Articles are most commonly published in the *Journal of Medical Internet Research* (6.8% [28/414]) and the JMIR sister journals: *JMIR Research Protocols*, *JMIR Mental Health*, *JMIR Public Health and Surveillance*, and *JMIR Medical Education* (combined 2.4% [10/414]). The next most common journals were *PLoS One* (2.4% [10/414]), *American Journal of Bioethics* (2.2% [9/414]), *AIDS and Behavior* (1.4% [6/414]), and *Nurse Researcher* (1.4% [6/414]). The remaining journals published 5 or fewer social media papers each. Nearly half of the studies published were empirical (42.8% [177/414]), followed by commentaries or opinion pieces (26.3% [109/414]) and conference abstracts (12.6% [52/414]). Other types of papers included discussion papers; theoretical, ethical, or methodological papers; literature reviews; and dissertations.

First authors of included articles represented 31 different countries, most commonly the United States (52.7% [218/414]), the United Kingdom (11.4% [47/414]), Australia (9.4% [39/414]), and Canada (7.0% [29/414]). The remaining countries of origin are shown in Table 1. The health discipline of the first authors varied, with the most common being medicine (33.3% [138/414]), nursing (10.9% [45/414]), public health (7.7% [32/414]), and psychology (3.6% [15/414]). First author discipline was unclear or not specified for 60 articles (14.5% [60/414]), and 30 articles (7.2% [30/414]) pertained to disciplines outside the health field, including but not limited to communication studies, journalism, law, and information studies.

**Table 1:** Country of first author (N=414).

Country of first author	Value, n (%)
United States	218 (52.7)
United Kingdom	47 (11.4)
Australia	39 (9.4)
Canada	29 (7)
Unknown	28 (6.8)
Germany, Saudi Arabia	6 (1.4)
Brazil, Italy, New Zealand, Spain	4 (1)
Ireland	3 (0.7)
Hong Kong, India, Israel, Norway, the Netherlands	2 (0.5)
Chile, Denmark, Finland, France, Iran, Japan, Northern Ireland, Singapore, South Africa, Sweden, Uganda	1 (0.2)
Multiple countries (cowritten article)	1 (0.2)

### Area of Health Research

A third of the articles included were nonspecific and covered health broadly (33.1% [137/414]), touching on 61 different health areas. Predominant topics included infectious diseases (eg, West Nile, Ebola, Zika, and HIV, 7.2% [30/414]), substance use (eg, smoking, alcohol, and marijuana, 6.8% [28/414]), cancer (6.5% [27/414]), mental health (eg, depression and anxiety, 5.3% [22/414]), and chronic disease (eg, diabetes and dementia, 4.6% [19/414]). The remainder of the topics were covered in fewer than 3% of studies.

### Purpose for Social Media Use

A quarter of the articles used social media for purposes of participant recruitment (25.1% [104/414]), followed by discussion on practical ways to use social media (15.5% [64/414]) or for content analysis (eg, the frequency and content of tweets on a certain topic; 13.3% [55/414]). Table 2 outlines the full list of uses for social media covered in the papers.

**Table 2:** Social media purpose (N=414).

Social media purpose	Value, n (%)
Participant recruitment	104 (25.1)
Practical use of social media	64 (15.5)
Content analysis	55 (13.3)
Promotion of academic research	43 (10.4)
Ethics and ethical concerns	33 (8.0)
Data mining from social media	26 (6.3)
Intervention or campaign implementation	26 (6.3)
Engagement of knowledge users	15 (3.6)
Conference tweeting	14 (3.4)
Research education (virtual journal clubs)	12 (2.9)
Data collection from participants	6 (1.4)
Reporting of research findings	4 (1.0)
Accessing scientific resources	4 (1.0)
Crowdfunding	3 (0.7)
Patient education and care	3 (0.7)
Collaborator engagement	1 (0.2)
Information health management	1 (0.2)

### Social Media Platforms

In articles that used or discussed at least one specific social media platform, an average of 1.33 (SD 0.7) different platforms were specified; 101 (24.4% [101/414]) articles did not specify a specific social media platform. Of those that did specify a platform, the most common were Twitter (38.2% [158/414]), Facebook (34.8% [144/414]), blogs (8.2% [34/414]), YouTube (6.3% [26/414/]), LinkedIn (2.7% [11/414]), Instagram (1.9% [8/414]), or research websites, such as ResearchGate or Academia.edu (1.5% for both [6/414]). Other platforms identified were used fewer than 5 times, including but not limited to crowdfunding platforms (eg, GoFundMe, MySpace, Google+, and Pinterest). Table 3 outlines how health researchers are currently using each social media platform for research purposes. For example, most participant recruitment occurs on Facebook (81.7% [335/414]), whereas content analysis (54.5% [226/414]) and data mining (76.9% [318/414]) occurs on Twitter.

**Table 3:** Social media use by social media platform.

Social media use	Twitter, n	Facebook, n	Blogs^a^, n	YouTube, n	LinkedIn, n	Instagram, n	Research websites^b^, n	Other platforms, n	Platform not specified, n
Participant recruitment	20	85	4	1	—^c^	1	—	11	11
Practical use of social media	25	13	6	3	6	2	2	2	33
Content analysis	30	7	6	11	—	3	—	3	—
Promotion of academic research	18	7	14	1	2	—	4	1	13
Ethics and ethical concerns	3	7	1	—	1	—	—	—	24
Data mining from social media	20	—	—	4	—	—	—	1	2
Intervention or campaign implementation	10	15	1	3	1	—	—	2	2
Engagement of knowledge users	3	6	1	—	—	1	—	1	5
Conference tweeting	14	—	—	—	—	—	—	—	—
Research education (online journal clubs)	7	—	—	1	—	—	—	—	4
Data collection from participants	2	2	—	—	—	1	—	—	1
Reporting of research findings	3	1	1	—	—	—	—	—	1
Accessing scientific resources	2	—	—	—	—	—	—	—	1
Crowdfunding	—	—	—	—	—	—	—	3	—
Patient education and care	—	—	—	1	—	—	—	—	3
Collaborator engagement	1	1	—	1	—	—	—	—	—
Information health management	—	—	—	—	—	—	—	—	1

^a^WordPress and Tumblr.

^b^eg, ResearchGate.

^c^No data available.

### Sentiment Classification on Social Media Use

**Figure 3:**
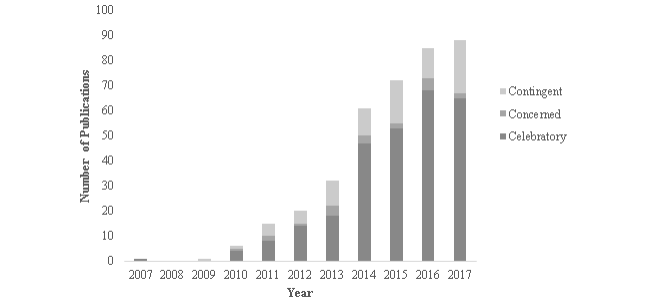
Classification of publications by year.

As outlined in Table 4, included articles were most commonly categorized using the Nettleton et al [27] sentiment classification as celebratory, (72.2% [299/414]), followed by contingent (22.7% [94/414]) and concerned (5.1% [21/414]). Articles classified as contingent or concerned were predominantly focused on the ethics of social media or the use of social media for content analysis. With the exception of articles focused on social media ethics (15.2% [63/414]) and use for content analysis (58.2% [241/414]), the remaining articles were predominantly classified as celebratory. Figure 3 plots the classification of articles over time, illustrating the relative consistency of perception over time.

**Table 4:** Reaction classification of the top 10 social media topics covered.

Social media topics	Celebratory, n (%)	Concerned, n (%)	Contingent, n (%)
Participant recruitment (n=104)	81 (77.9)	4 (3.8)	19 (18.3)
Practical use of social media (n=60)	48 (80)	2 (3)	10 (17)
Content analysis (n=55)	32 (58)	7 (13)	16 (29)
Promotion of academic research (n=43)	32 (77)	2 (5)	9 (21)
Ethics and ethical concerns (n=33)	5 (15)	5 (15)	23 (70)
Data mining from social media (n=26)	22 (85)	—^a^	4 (15)
Intervention or campaign implementation (n=26)	23 (89)	—	3 (11)
Engagement of knowledge users (n=17)	14 (82)	1 (6)	2 (12)
Conference tweeting (n=14)	12 (86)	—	2 (14)
Research education (online journal clubs; n=12)	11 (92)	—	1 (8)

^a^No data available.

## Discussion

### Principal Findings and Comparison With Prior Work

Results of this scoping review identified how health researchers are using social media for research purposes within 414 articles that met inclusionary criteria and were published after 2000 and before May 2018, with the first articles on this topic published in 2007. There has been substantial growth in the number of studies published on health researchers’ use of social media over the past decade, with the greatest increase occurring over the past 5 years. The increased interest in this field may reflect the widespread adoption of social media across academic contexts, including interest by journals [11], conferences [28], within institutions [9], and among individual scientists [29]. The number of published papers on social media will likely continue to rise, given its increasing popularity within academia.

The vast majority of publications originate from scholars in high-income countries, with 80.4% (333/414) of first authors based in the United States, the United Kingdom, Australia, or Canada. This is consistent with statistics on worldwide social media penetration that show North America ranking first with a social media penetration rate of 70%, followed by Northern Europe with 66% penetration [30]. The global average penetration rate reported in 2018 was 42%. A recent review found that the use of social media for health-related purposes is increasing in low- and middle-income countries [31], yet reliable access, cost, and infrastructure remain a barrier to the internet in some low- and middle-income countries. With regard to possible publication bias, an early study by Man et al [32] identified that research spending and English proficiency were associated with a greater likelihood of having publication output in high-ranking medical journals, which may have influenced the low number of articles identified from low- and middle-income countries. The higher rate of social media publications stemming from North America was also observed in a review published in 2014, in which the authors expected to see a rise in studies from low- and middle-income countries *in the future* [18]. However, it appears that the landscape has not yet shifted. This is intriguing, given that one of the main advantages of social media lies in its potential to disseminate research evidence with greater reach and at a relatively low cost [33]. Future research should explore the use, opportunities, and barriers of social media use by health researchers based on available technology (ie, bandwidth and hardware), research funding structures, geography, and socioeconomic factors (eg, gender, race, education, and income).

This study revealed that health researchers use social media for a range of research-related purposes. Most commonly, social media is used to recruit participants and to source data from the Web (eg, content analysis of social media posts and data mining on social media). Social media appears to facilitate research on clinical populations who have traditionally been difficult to recruit or study because of stigmatization, social disadvantage, low disease prevalence, or mobility challenges that make physical participation difficult [34]. In this review, we see this reflected in the health areas most associated with social media use, including infectious disease, substance use, cancer, mental health, and chronic disease. The other topics covered in the papers identified were practical use of social media and ethical concerns related to social media use. Articles identified various practical ways to use social media, including but not limited to how to incorporate social media into clinical trials, how to use social media to advance careers, how to use social media to disseminate research findings, and how to use various social media platforms. Similarly, ethical concerns arose related to various topics, including but not limited to social media in clinical trials, privacy concerns, professional relationships, and use of social media as a recruitment tool.

Most of the studies identified in this review used social media in primarily passive ways (eg, for participant recruitment or content analysis), with more active application noted in a handful of studies where it was used to support intervention delivery, promote campaigns (6.3% [26/414]), or build knowledge user engagement (3.6% [15/414]). Health researchers are not yet harnessing the full range of benefits available through social media, and this may reflect their lack of being social media savvy with regard to platform functions, audiences, features, or best practices. Health researchers are also using social media channels to promote their research, a practice that has been associated with increased article views and downloads [35]. Whether promoting articles over social media translates to an increase in citations remains unclear [14,36,37], as does the relationship between social media promotion and traditional academic metrics [38].

Health researchers rarely specify the social media platform used for research purposes, but when they do, they favor Twitter and Facebook. This is largely consistent with what has been reported previously [39-41]; however, this could be related to a function of historical emergence, whereby platforms such as Instagram and Snapchat became available later, and their use has not yet been widely reported in peer-reviewed literature. Health research using these newer platforms is still evolving, and it remains to be seen if it will prove to be useful for research purposes; fewer than 2% of the studies in this review discussed Instagram and none mentioned Snapchat. There is no *one-size-fits-all* platform for research-based social media use because use depends on purpose [42]. For example, Twitter may be effective for connecting with other scientists, but researchers seeking to reach adolescents and young adults should consider YouTube, Instagram, and Snapchat, as these are currently the most popular platforms among this age group [42]. The rapidly evolving social media landscape poses a challenge for health researchers given the significantly slower pace of empirical research and evaluation; some platforms that were popular just a few years ago (eg, MySpace) are now obsolete. This challenge is common among electronic health (eHealth) tools more generally [43] and not unique to social media. Baker et al [44] provide recommendations for conducting eHealth research, including social media, to optimize its timeliness and, in turn, its usefulness and effectiveness.

Health researcher sentiment regarding social media use was mostly celebratory, with a smaller percentage of researchers reporting concern and hesitation related to the ethical use of social media for research and analysis of social media content from online forums, Facebook groups, and Twitter hashtag conversations and comments. These apprehensions are not new; the ethics of conducting research using online communities has been a contentious area of debate over the past two decades [45]. The controversy lies in whether social media content is public or private information and extends to issues around confidentiality, informed consent, voluntary participation, and the potential for harm for both vulnerable populations and for researchers [3,46-48]. For health researchers who are also regulated professionals, social media may pose additional challenges related to patient privacy, maintaining professional boundaries, and the potential for misinterpretation of medical information [49]. In response, some organizations have developed policies to guide social media use among researchers [50-52]. However, there is no common standard policy or procedure to guide social media use in health research.

### Limitations

Although this scoping review was conducted according to scoping review methodology, there were some limitations that are worth noting. Data were extracted by only 1 reviewer owing to the high number of studies identified in the data extraction phase. To minimize error, 2 reviewers identified relevant studies, and a standardized extraction form was used to ensure accuracy. As the data extracted were descriptive and did not include study results, the impact of potential data extraction errors is minimal.

We defined health researcher broadly, including the spectrum of bench to bedside. This resulted in a wide search strategy yielding over 8000 initial titles to screen. Social media practices likely vary, such that clinical health researchers working with vulnerable populations on the Web may be more inclined to recognize ethical challenges as compared with a public health researcher seeking to disseminate evidence on the latest flu vaccination. However, broad inclusion of a range of health researchers enabled us to gain a wide-ranging picture of current social media practices, thereby increasing the external validity of our findings.

A final limitation is the rapid growth of the field, whereby challenges with currency of publication is noted. This field is moving rapidly, so it is important to acknowledge that this scoping review is a snapshot at a particular point in time.

### Conclusions

In conclusion, health researchers are increasingly using social media for a range of professional purposes, and the evidence reflecting this use varies widely. Although most of the sentiment around the use of social media in health research is celebratory, there are concerns about the ethics of social media use for some purposes. Future initiatives are needed to support health researchers to navigate the social media landscape and evaluate the impact of their efforts. Given the concerns related to ethics and content analysis of social media, future work should focus on providing additional direction to health researchers on how to ethically use and engage with social media. This could include the development of professional or institution-specific guidelines or the development of best practices.

## Appendix

Multimedia Appendix 1 Search strategies for electronic databases.

## Abbreviations

eHealth: electronic health
PRISMA: Preferred Reporting Items for Systematic Reviews and Meta-Analyses
