# Supplementary material for: Health Researchers’ Use of Social Media: Scoping Review
Source: J Med Internet Res. 2019 Nov 13;21(11):e13687. doi: 10.2196/13687 (PMC6881779; doi:10.2196/13687)
Supplement: Multimedia Appendix 1 [file jmir_v21i11e13687_app1.pdf]

Multimdia Appendix 1: Search strategies for electronic databases

MEDLINE (Ovid, 1946 to May 23, 2018)

#	Searches
1	((altmetric* or linkedin or 'linked in' or 'social network*' or 'social media' or 'social medium*' or twitter or tweet* or facebook or 'web 2*' or instagram* or snapchat* or blog* or weblog* or tumblr or youtube or vimeo) adj5 ('research recruit*' or 'study recruit*' or 'participant recruit*' or researcher* or academic* or scholar* or scientist* or investigator* or research)).ti,ab,kw.
2	exp social media/
3	exp Social Networking/
4	exp research/
5	exp research personnel/
6	exp ethics, research/
7	4 or 5 or 6
8	2 or 3
9	7 and 8
10	1 or 9

Embase (Elsevier/Embase.com, 1947 to May 23, 2018)

No.	Query
#5	(('scientist'/exp OR 'research'/exp) AND 'social media'/exp) OR ((altmetric* OR linkedin OR 'linked in' OR 'social network*' OR 'social media' OR 'social medium*' OR twitter OR tweet* OR facebook OR 'web 2.0' OR instagram* OR snapchat* OR blog* OR weblog* OR tumblr OR youtube OR vimeo) NEAR/5 ('research recruit*' OR 'study recruit*' OR 'participant recruit*' OR researcher* OR academic* OR scholar* OR scientist* OR investigator* OR research)):ti,ab,kw
#4	((altmetric* OR linkedin OR 'linked in' OR 'social network*' OR 'social media' OR 'social medium*' OR twitter OR tweet* OR facebook OR 'web 2.0' OR instagram* OR snapchat* OR blog* OR weblog* OR tumblr OR youtube OR vimeo) NEAR/5 ('research recruit*' OR 'study recruit*' OR 'participant recruit*' OR researcher* OR academic* OR scholar* OR scientist* OR investigator* OR research)):ti,ab,kw
#3	('scientist'/exp OR 'research'/exp) AND 'social media'/exp
#2	'social media'/exp
#1	'scientist'/exp OR 'research'/exp

CINAHL, inception 1971, and PsycINFO, inception 1967 (EbscoHost, inception to May 27, 2018)

#	Query
S10	S1 OR S9
S9	S7 AND S8
S8	S2 OR S3
S7	S4 OR S5 OR S6
S6	DE "Social Media" OR DE "Online Social Networks" OR DE "Online Social Networks" OR DE "Blog"
S5	(MH "Social Networking")
S4	(MH "Social Media")
S3	DE "Experimentation" OR DE "Action Research" OR DE "Animal Research" OR DE "Consumer Research" OR DE "Interdisciplinary Research" OR DE "Online Experiments" OR DE "Qualitative Research" OR DE "Quantitative Methods" OR DE "Research Setting" OR DE "Experimental Ethics" OR DE "Experimental Recruitment" OR DE "Experimenters"
S2	(MH "Research+")
S1	TI (((altmetric* OR linkedin OR "social network*" OR "social media" OR "social medium*" OR twitter OR tweet* OR facebook OR "web 2*" OR instagram* OR snapchat* OR blog* OR weblog* OR tumblr OR youtube OR vimeo) N5 ("research recruit*" OR "study recruit*" OR "participant recruit*" OR researcher* OR academic* OR scholar* OR scientist* OR investigator* OR research))) OR AB (((altmetric* OR linkedin OR "social network*" OR "social media" OR "social medium*" OR twitter OR tweet* OR facebook OR "web 2*" OR instagram* OR snapchat* OR blog* OR weblog* OR tumblr OR youtube OR vimeo) N5 ("research recruit*" OR "study recruit*" OR "participant recruit*" OR researcher* OR academic* OR scholar* OR scientist* OR investigator* OR research)))

ERIC (Proquest, 1966 to May 27, 2018)

noft(((altmetric* OR linkedin OR "social network*" OR "social media" OR "social medium*" OR twitter OR tweet* OR facebook OR "web 2*" OR instagram* OR snapchat* OR blog* OR weblog* OR tumblr OR youtube OR vimeo) NEAR/5 ("research recruit*" OR "study recruit*" OR "participant recruit*" OR researcher* OR academic* OR scholar* OR scientist* OR investigator* OR research))) AND noft(health OR medicine OR medical OR healthcare OR nursing OR nurse* OR dentist* OR pharmac* OR physician* OR doctor* OR surger* OR surgical*)

Web of Science Core Collection (Clarivate, 1900 to May 27, 2018)

TS=(((altmetric* OR linkedin OR "social network*" OR "social media" OR "social medium*" OR twitter OR tweet* OR facebook OR "web 2*" OR instagram* OR snapchat* OR blog* OR weblog* OR tumblr OR youtube OR vimeo) NEAR/5 ("research recruit*" OR "study recruit*" OR "participant recruit*" OR researcher* OR academic* OR scholar* OR scientist* OR investigator* OR research))) AND TS=((health OR medicine OR medical OR healthcare OR nursing OR nurse* OR dentist* OR pharmac* OR physician* OR doctor* OR surger* OR surgical*))
